# Easy Wound Bed Preparation by Polyacrylate Pad with Silver Matrix and Curettage

**DOI:** 10.1097/GOX.0000000000001954

**Published:** 2018-09-24

**Authors:** Apinut Wongkietkachorn, Palakorn Surakunprapha, Suapa Theeragul

**Affiliations:** From the * Department of Surgery, Faculty of Medicine, Mae Fah Luang University, Chiang Rai, Thailand; † Department of Surgery, Faculty of Medicine, Khon Kaen University, Khon Kaen, Thailand.

## Abstract

Supplemental Digital Content is available in the text.

Dealing with chronic wounds and biofilm require a great deal of surgeon’s persistence and patience. Once biofilm has formed, it is traditionally removed by curettage or debridement, which can be difficult due to the patient’s pain and bleeding.^1^ As an alternative, we propose applying a polyacrylate pad with silver matrix (UrgoClean Ag; Urgo Healthcare Product, France), and then opening it and curetting the wound daily. A polyacrylate pad is used because of its unique feature that can make the curettage easy with minimal bleeding and minimal damage to the granulation tissue.^2^ An example is shown in Figures 1 and 2, and the process is illustrated in SDC 1. (See video, Supplemental Digital Content 1, which displays easy wound bed preparation, available in the “Related Videos” section of the Full-Text article on PRSGlobalOpen.com or at http://links.lww.com/PRSGO/A870). The comparison is demonstrated by using t raditional wet-to-dry gauze dressings and curettage, which is shown in SDC 2. (See **figure, Supplemental Digital Content 2**, which displays result after application of traditional wet-to-dry gauze dressings and curettage. There were some blot clots as a result of some bleeding from the wound bed, http://links.lww.com/PRSGO/A871).

**Fig. 1.**
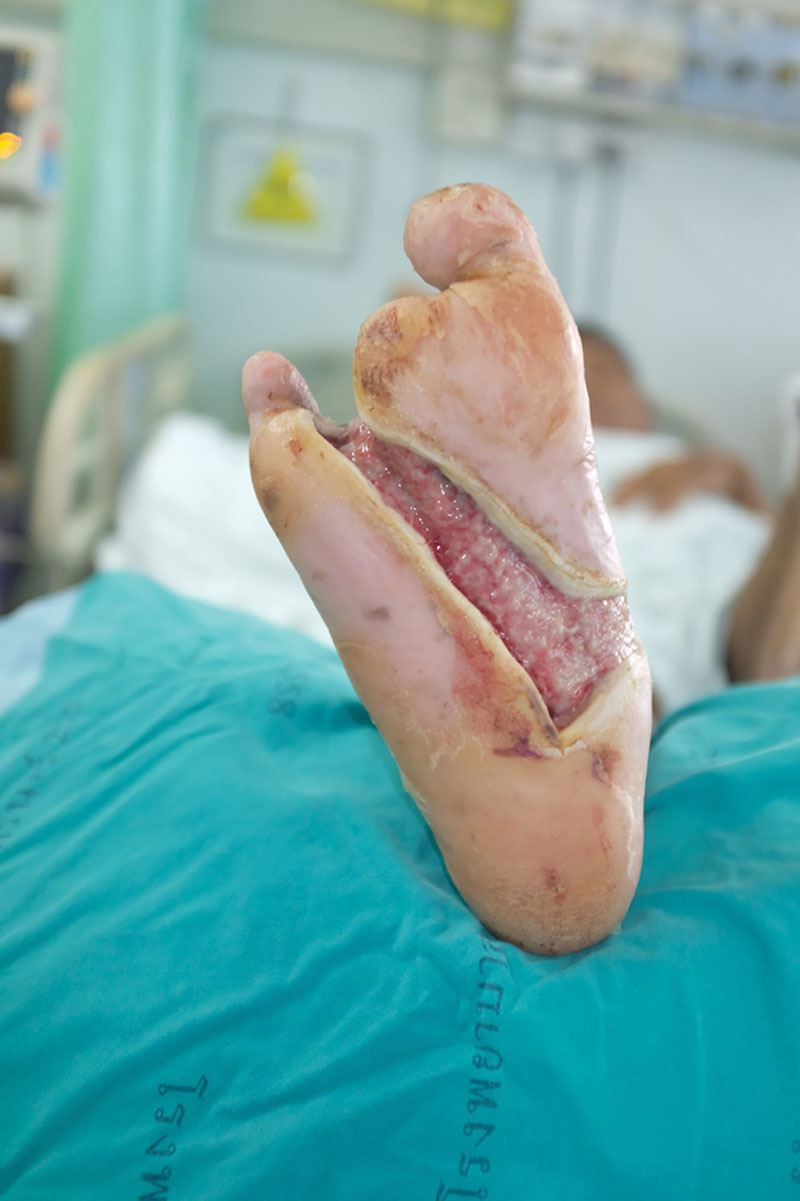
Chronic infected diabetic ulcer on the right foot with slough and biofilm.

**Fig. 2.**
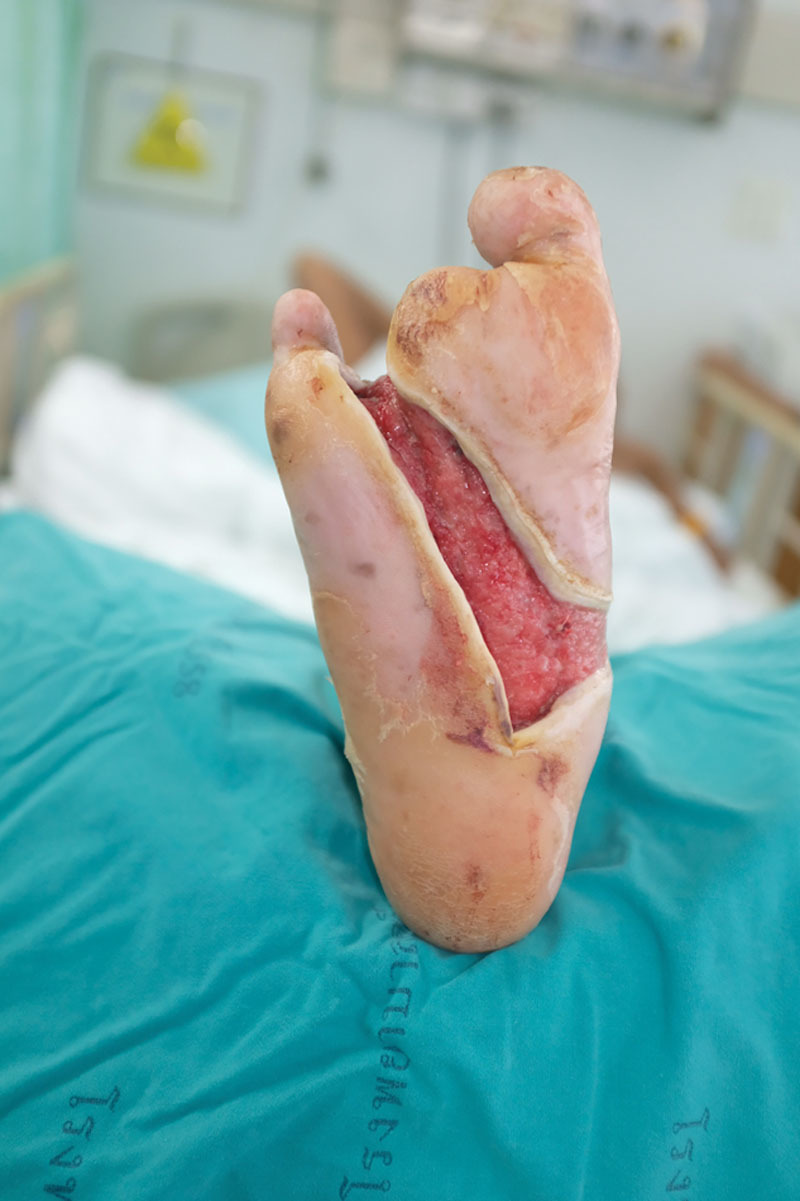
Result after application of a polyacrylate pad with silver matrix for 1 day and curettage. The wound was clean without significant bleeding. Later, the wound was covered with split-thickness skin graft and yielded good graft take.

**Video Graphic 1.**
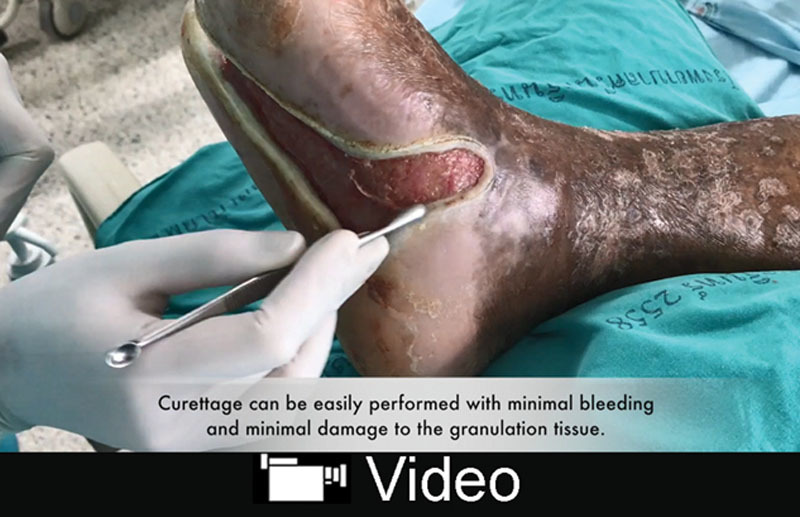
See video, Supplemental Digital Content 1, which displays easy wound bed preparation. This video is available in the “Related Videos” section of the Full-Text article on PRSGlobalOpen.com or at http://links.lww.com/PRSGO/A870.

The easy wound bed preparation comes from the fact that the polyacrylate fiber helps disrupt the structural integrity of biofilm 24 hours after application, making curettage easier. In addition, silver matrix is composed of silver ions that provide antimicrobial activity and lipido-colloid components that help prevent wound adherence to the dressing during removal, which can occur in traditional wet-to-dry gauze method.^2^ We hope that the method we describe can be used as a lower risk alternative and less-aggressive way for preparing wound beds.

## Supplementary Material

## References

[1] Schultz GS Sibbald RG Falanga V Wound bed preparation: a systematic approach to wound management. Wound Repair Regen. 2003;11:S1 28. 12654015 10.1046/j.1524-475x.11.s2.1.x

[2] Desroche N Dropet C Janod P Antibacterial properties and reduction of MRSA biofilm with a dressing combining polyabsorbent fibres and a silver matrix. J Wound Care. 2016;25:577 584. 27681588 10.12968/jowc.2016.25.10.577

