# Supplementary figures and images for: Easy Wound Bed Preparation by Polyacrylate Pad with Silver Matrix and Curettage
Source: Plast Reconstr Surg Glob Open. 2018 Sep 24;6(9):e1954. doi: 10.1097/GOX.0000000000001954 (PMC6191242; doi:10.1097/GOX.0000000000001954)

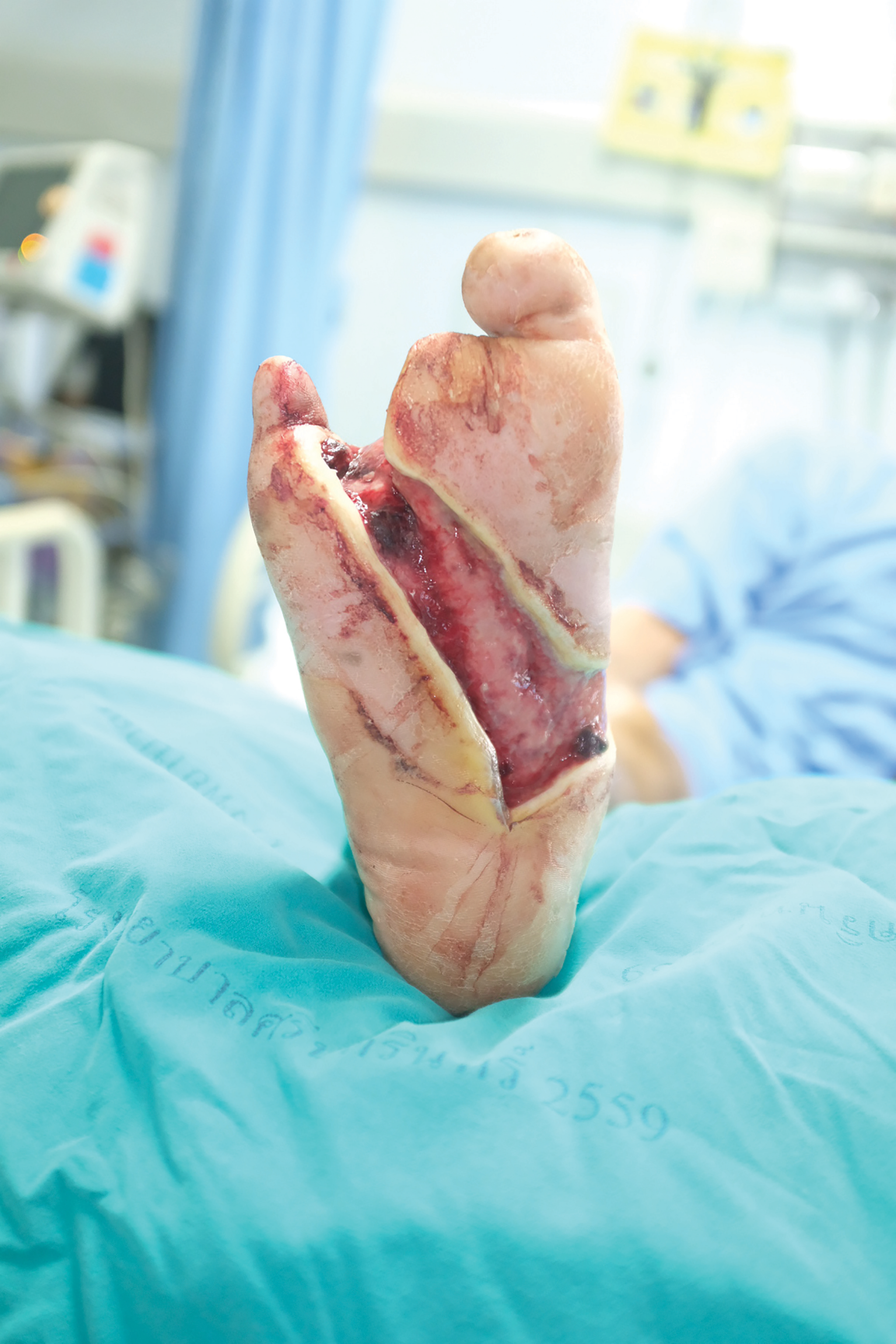

โรงพยาบาลศิริราช วันที่ 2559

Supplement: Supplementary file 2 [file gox-6-e1954-s002.pdf]
